# Book Reviews

**Published:** 1896-09

**Authors:** 


### Book Reviews

A MANUAL OF MEDICAL JURISPRUDENCE AND TOXICOLOGY. By Henry C. Chapman, M. D., Professor of Institutes of Medicine and Medical Jurisprudence in the Jefferson Medical College of Philadephia, etc. Second edition, revised, with 55 illustrations and 3 plates in colors. Philadelphia: W. B. Saunders, 1896. Price $1.50. Pp. 254.

The second edition of this valuable work has been thoroughly revised and new material added. The author's experience as Coroner's physician in Philadelphia makes his work of additional value. Some new figures have been added. The cut illustrating the fœtal circulation is particularly good. Among the smaller works on this subject, Chapman's is by far the best.

---

A MANUAL OF ANATOMY. By Irving S. Haynes, Ph. B., M. D., Adjunct Professor and Demonstrator of Anatomy in the Medical Department of the New York University. With 134 Half-tone Illustrations and 42 Diagrams. Philadelphia: W. B. Saunders, 925 Walnut Street. 1896. Price, $2.50 net.

This manual is to be highly endorsed as a practical guide. The illustrations of dissections are from photographs made by the author, and are sufficiently numerous to elucidate the text. The dissections of the abdomen, the layers of muscles of the sole of the foot, sections of the brain, are particularly instructive. The introduction of photographs into a book of this character marks an incalculable advance over the older cuts, however good they were.

---

DIAGNOSIS AND TREATMENT OF DISEASES OF THE RECTUM, ANUS, AND CONTIGUOUS TEXTURES. Designed for practitioners and students. By S. W. Gant, M. D. With two chapters on "Cancer" and "Colotomy," by Herbert William Allingham, F. R. C. S., Eng. Royal 8vo., pp. 400. Illustrated with 16 full page chromo lithographic plates, and 115 wood engravings in the text. Philadelphia. The F. A. Davis Co. Price, extra cloth, $3.50 net: half russia, gilt top, $4.50, net.

This book has several points for favorable consideration; its brevity and succinctness, the accuracy of its illustrations and the chapter on "Railroading as an Etiological Factor in Rectal Disease," and "Auto-Intoxication, or Auto-Infection from the Intestinal Canal." The chapters on "Colotomy and Cancer," by Dr. Allingham, need no commendation, as the work of this distinguished English surgeon is already so well known. The style of the book is clear and direct, but by no means elegant; the homely expressions serve to give force to the idea rather than take from it. The illustrations are graphic and excellent, worthy reproductions of photographs.

---

THE THREE ETHICAL CODES. That of the American Medical Association; its Constitution, By-Laws, Amendments, etc. That of the American Institute of Homœopathy, and that of the National Eclectic Medical Society. Limp cloth, round corners, 55 pages, postpaid 50 cents. The Illustrated Medical Journal Co., Publishers, Detroit, Mich.

By comparing the Code of the Homœopathic Society with that of the American Medical Association, it will be found that several sections of the former are similar to the latter's code. The Eclectic Code is worthy of mention for its brevity.

